# Exposure to Radiation and Thyroid Cancer Risk Among Young Female Nurses: Longitudinal Analysis From the Korea Nurses’ Health Study

**DOI:** 10.2196/68037

**Published:** 2025-09-18

**Authors:** Young Taek Kim, Choa Sung, Yanghee Pang, Chiyoung Cha

**Affiliations:** 1 Division for Gender-based Violence Research Korean Women's Development Institute Seoul Republic of Korea; 2 Translational Biobehavioral and Health Promotion Branch National Institute of Health Clinical Center Bethesda, MD United States; 3 Department of Nursing Seoil University Seoul Republic of Korea; 4 College of Nursing Ewha Womans University Seoul Republic of Korea

**Keywords:** cohort studies, occupational exposure, longitudinal studies, nurses, thyroid neoplasms, women’s health

## Abstract

**Background:**

Thyroid cancer is one of the most commonly diagnosed malignancies in South Korea, with incidence rates among the highest globally. Young women, in particular, represent a high-risk group, likely due to a combination of biological, occupational, and environmental factors. However, the specific risk factors contributing to thyroid cancer development in this population remain poorly understood.

**Objective:**

This study aims to identify the risk factors associated with thyroid cancer among young female nurses using longitudinal survival analysis.

**Methods:**

This longitudinal study used data from the Korea Nurses’ Health Study (KNHS), a prospective national cohort of female nurses. Data from the first, fifth, seventh, and ninth surveys were used to construct a person-period data set. Female nurses aged in their 20s at baseline were included. Time-varying explanatory variables included age, marital status, BMI, smoking, alcohol consumption, perceived stress, sleep problems, nursing position, night shift work, working unit, and duration of radiation exposure. The dependent variable was self-reported physician-diagnosed thyroid cancer. Kaplan-Meier survival analysis and Cox proportional hazards regression were performed to examine the association between risk factors and thyroid cancer occurrence.

**Results:**

A total of 22,759 person-period cases were analyzed, and 105 thyroid cancer events were identified. Kaplan-Meier analysis showed significant associations between thyroid cancer occurrence and age (*χ*²_1_=51.6, *P*<.001), marital status (*χ*²_1_=25.1, *P*<.001), sleep problems (*χ*²_1_=20.3, *P*<.001), night shift work (*χ*²_1_=20.1, *P*<.001), working unit (*χ*²_1_=13.0, *P*<.001), and duration of radiation exposure (*χ*²_1_=91.0, *P*<.001). In the Cox regression model, nurses aged in their 20s had a significantly higher risk of thyroid cancer than those aged in their 30s (hazard ratio [HR] 4.602, 95% CI 1.893-11.188). Those who worked night shifts were also at an increased risk (HR 1.923, 95% CI 1.127-3.280). Compared with no exposure, radiation exposure showed a dose-response relationship: <1 year: HR 3.449, 95% CI 1.474-8.074; ≥1 year: HR 4.178, 95% CI 2.702-6.461.

**Conclusions:**

Younger age, night shift work, and duration of radiation exposure were significantly associated with an increased risk of thyroid cancer in young female nurses. These findings highlight the importance of early screening and occupational risk management, including regular radiation monitoring and support for circadian health, in health care settings.

**International Registered Report Identifier (IRRID):**

RR2-10.4178/epih.e2024048

## Introduction

In recent decades, thyroid cancer (TC) has emerged as the most rapidly increasing type of cancer worldwide, ranking ninth in incidence in the United States in 2020 [1]. The reported incidence rate of TC has doubled in the United States, rising from 7.62 per 100,000 people in 2000 to 13.83 per 100,000 in 2017, partly due to advancements in diagnostic technology [2]. Although TC has the highest 5-year survival rate among all cancers (98.3%), it imposes a substantial burden on the health care system and affected individuals due to psychological and financial challenges, especially given its recurrence rate of 4.3%-13.6% [3,4]. Among the global increase in TC incidence, South Korea reported especially high rates, ranging from 6.5 per 100,000 in 1999 to 40.2 per 100,000 in 2018—the highest reported values globally [5].

Notably, women in South Korea had higher age-standardized incidence (62.2 per 100,000 for women vs 18.8 per 100,000 for men) and mortality (0.4 per 100,000 for women vs 0.2 per 100,000 for men) in 2018 [6]. Specifically, women aged 15 years to 34 years are considered an at-risk group, accounting for 50% of all cancers diagnosed in South Korea [6]. TC is of particular concern for young adults aged in their 20s, as it is the second most commonly diagnosed cancer during pregnancy and up to 1 year postpartum [7]. Although concerns have been raised about overdiagnosis of TC—especially in South Korea, where improved access to health care has increased tumor detection rates [8]—and despite the low mortality rate of TC, all cancer survivors are subject to lifelong surveillance for disease recurrence. Furthermore, it is necessary to consider the changes associated with modern industrialized lifestyles, such as increased exposure to medical radiation, along with environmental and behavioral factors, which may serve as carcinogenic risk factors contributing to TC occurrence in young adult women [9].

As the etiology of TC remains poorly understood, researchers have explored potential risk factors to facilitate the development of preventive strategies. The most widely recognized contributor to the development of TC is exposure to ionizing radiation, and this risk is aggravated when individuals are exposed in their younger years [9]. Globally, 7.35 million medical professionals, including nurses, are exposed to occupational low-dose ionizing radiation, which may be associated with TC development [10]. However, the literature remains inconclusive, with conflicting findings on the statistical significance of the association between radiation exposure and TC risk among nurses [9,11]. Moreover, being female has been historically associated with an increased risk of papillary TC, likely due to the influence of reproductive hormones such as estrogen and progesterone [12]. As with all other cancer types, a family history of the disease—particularly the nonmedullary type—also contributes to the risk because genetic mutations increase an individual’s susceptibility to tumor formation [13,14].

Sociodemographic and lifestyle factors have long been the focus of research aimed at TC prevention in the general population. Large-scale cohort studies have demonstrated an association between obesity—typically measured via BMI—and TC risk [15], particularly among women, regardless of geographical or age differences [16]. Moreover, sleep disturbances have been associated with TC incidence. For example, a systematic review reported that night shift work may affect thyroid-stimulating hormone levels, which may contribute to the development of thyroid malignancies [17]. Traditional cancer risk factors such as cigarette smoking, alcohol consumption, and stress have also been investigated as risk factors for TC; however, the findings have been inconsistent. These discrepancies underscore the importance of exploring multiple lifestyle factors that may be implicated in TC risk [18].

Considering the inconsistent results regarding the potential risk factors for TC occurrence and the high incidence of TC observed among young adult Korean women, further investigation of these factors is needed to better understand their contribution to TC in women. Therefore, leveraging a large longitudinal data set from the ongoing Korea Nurses’ Health Study (KNHS), we investigated the risk factors that may be predictors of TC among female nurses aged in their 20s. We believe that the TC risk factors identified in this well-characterized population will be broadly representative of those identified in all South Korean women.

## Methods

To enhance the transparency and quality of observational studies, this study followed the STROBE (Strengthening the Reporting of Observational Studies in Epidemiology) checklist for cohort studies.

### Data Source and Study Population

**Figure 1:**
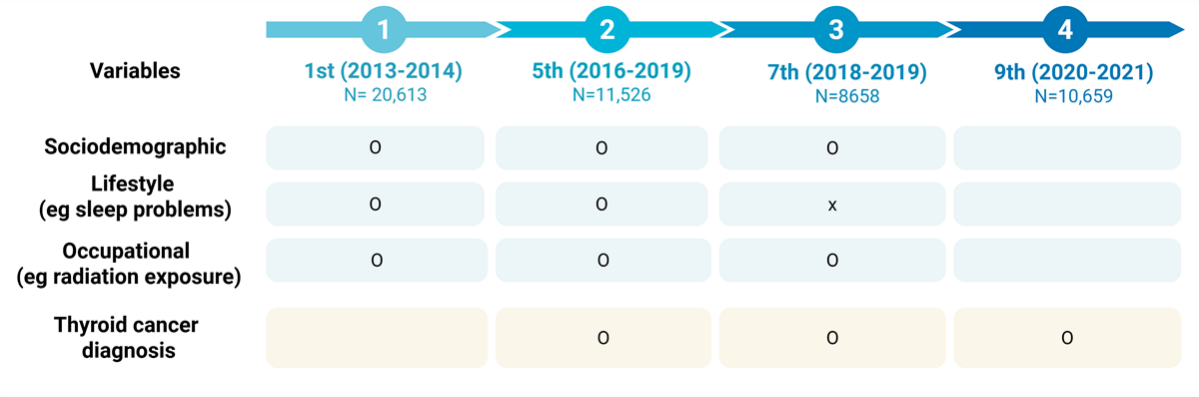
Measurement timeline across the Korea Nurses’ Health Study. Created with BioRender [20].

**Figure 2:**
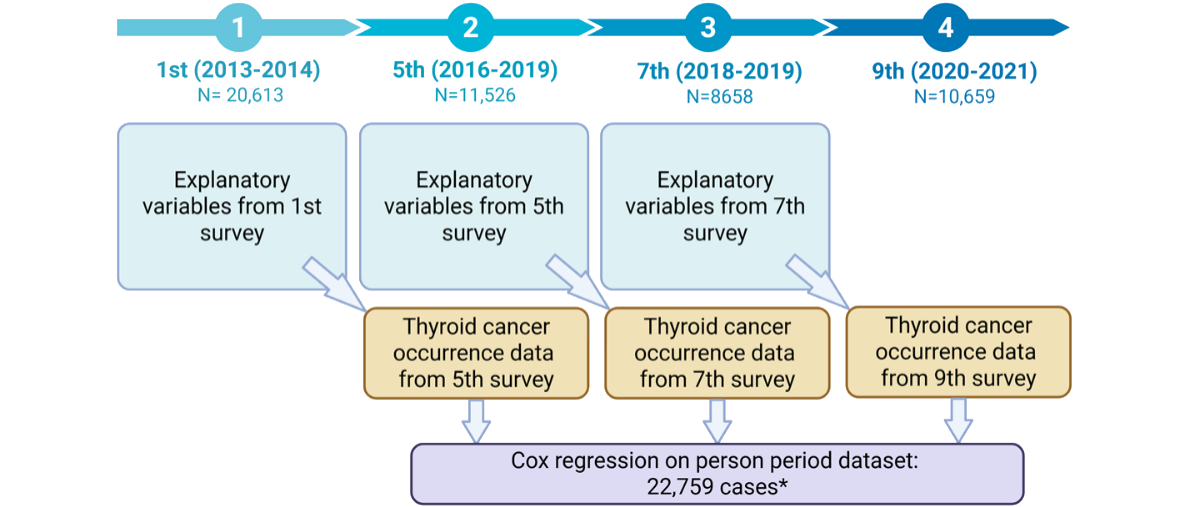
Person-period data structure. *Cases with missing data on explanatory or dependent variables were excluded by default. Created with BioRender [20].

In this study, we used data from the KNHS, an ongoing prospective cohort study designed to investigate the determinants of women’s health and illness. The initial KNHS survey was conducted from 2013 to 2014, enrolling 20,613 female nurses. Participants have since been followed through online surveys [19]. This analysis explored the influence of sociodemographic, lifestyle, and occupational characteristics on the occurrence of TC over time, using data from the first and fifth surveys in 2016-2019 (n=11,526), seventh survey in 2018-2019 (n=8658), and ninth survey in 2020-2021 (n=10,659). These 4 surveys were selected for the longitudinal analysis due to their high number of participants and consistency in question items. From the first survey, we identified a subset of women aged in their 20s (n=12,055). An overview of the measurement timeline across the surveys and the survival analysis structure are shown in Figures 1 and 2, respectively.

In longitudinal data sets, explanatory variables should precede the occurrence of a dependent variable to establish potential causal relationships between these variables. Accordingly, in this study, TC occurrence in the fifth survey was analyzed in relation to explanatory variables from the first survey, TC occurrence in the seventh survey was analyzed based on explanatory variables from the fifth survey, and TC occurrence in the ninth survey was analyzed based on explanatory variables from the seventh survey. This design allowed for the assessment of how time-varying explanatory variables influence TC occurrence (a dependent variable) over time.

The first, fifth, seventh, and ninth surveys were selected because the dependent variable—physician-diagnosed TC—was assessed in all but the second and sixth surveys. Additionally, the duration of radiation exposure in hospitals—a key explanatory variable for TC—was specifically addressed in the first, fifth, and seventh surveys.

### Measures

The dependent variable was defined as a dichotomized indicator of TC diagnosis made by a medical professional, as reported in the fifth, seventh, and ninth surveys. These surveys assessed the occurrence of TC before 2017, between 2018 and 2019, and between 2020 and 2021, respectively.

In this study, the explanatory variables were classified as sociodemographic, lifestyle, and occupational factors. Sociodemographic variables included age, marital status, and BMI in the first, fifth, and seventh surveys. Age was dichotomized as <30 years and ≥30 years. Marital status was categorized as married and unmarried/divorced/separated/widowed. BMI, calculated using height and weight, was categorized into underweight/normal (<23 kg/m^2^) and overweight or obese (23 kg/m^2^) groups.

Lifestyle variables included smoking status, alcohol consumption, perceived stress, and sleep problems. Smoking and alcohol consumption were assessed in the first and fifth surveys and categorized as yes or no. We analyzed whether smoking and alcohol consumption in the fifth survey could affect the occurrence of TC in the ninth survey because this information was not collected in the seventh survey. Perceived stress was measured on a 2-point scale. A 4-item perceived stress scale was used in the first survey, whereas a 10-item scale was used in the fifth and seventh surveys [21,22]. Both the 4-item and 10-item scales have been proven reliable and valid. The cutoff scores were determined based on the literature, with the top tertile categorized as high stress and the remaining tertiles as low stress [23]. The Cronbach α for the perceived stress scales was 0.52, 0.70, and 0.73 in the first, fifth, and seventh surveys, respectively. Sleep problems were measured using the Jenkins Sleep Scale [24]. Based on a cutoff point of 12 out of a total score of 20, sleep problems were dichotomized as yes or no. The Cronbach α for sleep problems was 0.88 and 0.83 in the first and fifth surveys, respectively. We analyzed whether sleep problems in the fifth survey could affect the occurrence of TC in the ninth survey because this information was not collected in the seventh survey.

Occupational variables included nursing position, night shift work, working unit, and duration of radiation exposure in the hospital during the first, fifth, and seventh surveys. Nursing positions were dichotomized into staff nurses, nursing unit managers, and administrative supervisors. Night shift work was categorized as yes or no. Working units were classified into general ward/outpatient ward/hospital administration or special care units (eg, intensive care or emergency, anesthesia, operating room). To assess radiation exposure in hospitals, we surveyed the exclusive use of X-ray rooms, portable X-rays, computed tomography scans, fluoroscopes, radioactive isotopes, and radioactive materials. The duration of radiation exposure was surveyed among patients who were exposed to radiation in the hospital. Responses were classified as no-exposure, <1 year, and ≥1 year.

### Statistical Analysis

Descriptive and cross-tabulation analyses were performed to compare sociodemographic, lifestyle, and occupational characteristics by TC status, using baseline data from the first survey (n=20,613). Longitudinal survival analysis was conducted using data from the first, fifth, seventh, and ninth surveys of the KNHS based on the literature, demonstrating that survival analysis can be performed using survey data with at least one follow-up [25]. Based on the findings of a previous study [26], explanatory variables in the first, fifth, and seventh surveys and the dependent variable TC in the fifth, seventh, and ninth surveys were used to construct a person-period data set. The data set was transformed into a long format with one row per person to capture temporal changes. After excluding cases with missing data on either explanatory or dependent variables (n=39,260), a total of 22,759 patients were included in the final analysis.

For the univariate analysis, a cumulative survival function test and Kaplan-Meier analysis were performed. The cumulative survival function test was performed to identify the effect of radiation exposure on TC occurrence. Kaplan-Meier analysis was performed to explore the relationship between each explanatory variable and the dependent variable, TC.

For the multivariate analysis, Cox regression analysis was conducted to identify the hazard ratios (HRs) of the explanatory variables affecting the dependent variable TC over time [25]. This approach accounted for the time-varying nature of explanatory variables and their influence on subsequent TC development. In multivariate analysis, there was no collinearity among the variables selected in the model. Furthermore, the Cox regression assumption—that the influence of independent variables on an event is constant over time—was confirmed [25].

### Ethical Considerations

This study was approved by the institutional review board overseeing the KNHS (approval number 2013-03CON-03-P). Detailed descriptions of KNHS data collection procedures are available in the study by Kim et al [19]. In brief, participants provided electronic informed consent after securely logging into the KNHS website using their names and nurse registration numbers. Consent included agreement to use de-identified survey data for research purposes, prospective follow-up through national databases, and the protection of personal information in compliance with Korea’s Privacy Protection Act. Additional consent was obtained for the use of contact information for follow-up and distribution of participation incentives. Survey data were anonymized for analysis, and personal identifiers were stored securely. Participants received a mobile gift card valued at approximately US $4.00 (₩4000) as compensation.

## Results

### Study Participant Characteristics

The results of the baseline descriptive analysis are presented in Table 1. This includes thyroid occurrence categorized by sociodemographic, lifestyle, and occupational variables.

**Table 1:** Study participant characteristics (n=20,613).

Variables				Participants, n		Thyroid cancer (yes), n (%)		Thyroid cancer (no) n (%)		*χ*^2^ (*df*)			*P* value	
**Sociodemographic variables**														
	**Age (years)**										66.1 (1)			<.001
		20-29	12,055		38 (0.3)		12,017 (99.7)							
		≥30	8558		110 (1.3)		8448 (98.7)							
	**Marital status**										41.6 (1)			<.001
		Married	13,646		61 (0.4)		13,585 (99.6)							
		Unmarried/divorced/separated/widowed	6965		87 (1.2)		6878 (98.8)							
	**Body mass index (kg/m^2^)**										8.0 (1)			.005
		<23	3886		41 (1.1)		3845 (98.9)							
		≥23	16,634		105 (0.5)		16,529							
**Lifestyle variables**														
	**Smoking**										0.007 (1)			.93
		Yes	671		5 (0.7)		666 (99.3)							
		No	19,938		143 (0.7)		19,795 (99.3)							
	**Alcohol consumption**										13.9 (1)			<.001
		Yes	11,999		66 (0.6)		11,933 (99.4)							
		No	6576		68 (1.0)		6508 (99)							
	**Perceived stress**										0.6 (1)			.44
		Low (≤11)	19,802		144 (0.7)		19,658 (99.3)							
		High (>11)	811		4 (0.5)		807 (99.5)							
	**Sleep problems**										1.0 (1)			.32
		Yes (≥12)	4005		24 (0.6)		3981 (99.4)							
		No (<12)	16,605		124 (0.7)		16,481 (99.3)							
**Occupational variables**														
	**Job positions**										26.2 (1)			<.001
		Staff nurse	17,044		101 (0.6)		16,943 (99.4)							
		Nursing unit manager/administrative supervisor	3227		46 (1.4)		3181 (98.6)							
	**Night shift work**										37.1 (1)			<.001
		Yes	13,995		66 (0.5)		13,929 (99.5)							
		No	6618		82 (1.2)		6536 (98.8)							
	**Working units**										7.2 (1)			.007
		General ward/outpatient ward/hospital administration	18,761		144 (0.8)		18,617 (99.2)							
		Special care units^a^	1852		4 (0.2)		1848 (99.8)							
	**Duration of exposure to radiation in hospital**										10.4 (2)			.006
		None	17,839		141 (0.8)		17,698 (99.2)							
		<1 year	343		2 (0.6)		341 (99.4)							
		≥1 year	2431		5 (0.2)		2426							

^a^Intensive care unit, emergency room, anesthetic room, and operating room.

### Cumulative Survival Analysis

**Figure 3:**
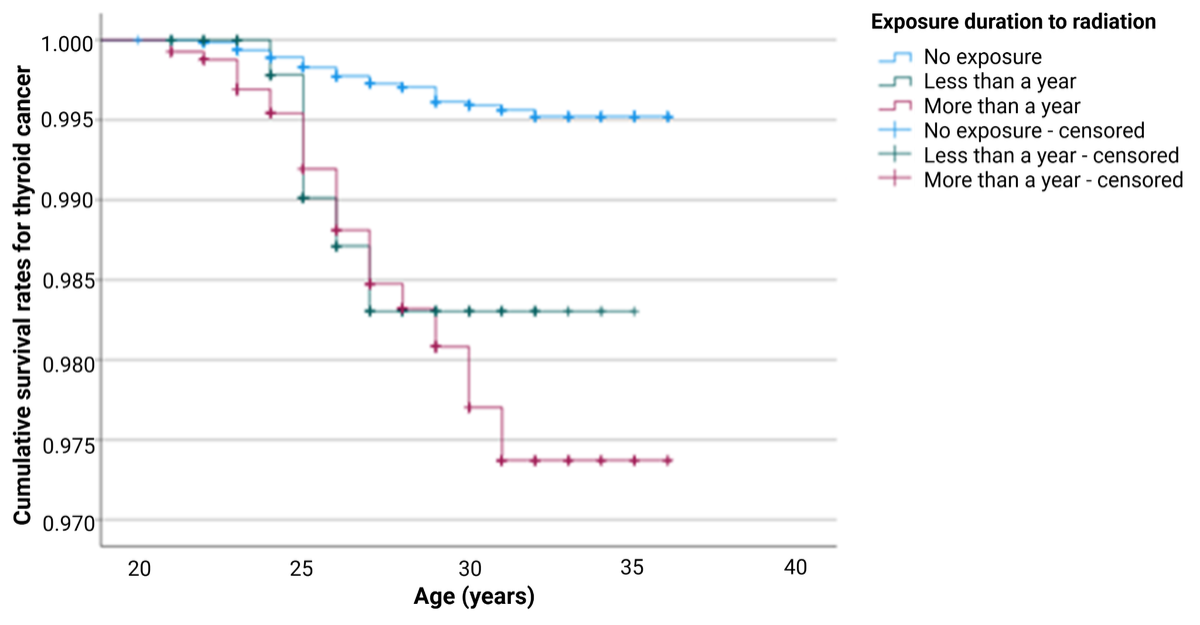
Cumulative survival rates for thyroid cancer by the duration of radiation exposure in the hospital. Created with BioRender [20].

The results of the cumulative survival function test for assessing the association between TC occurrence and duration of radiation exposure in the hospital are shown in Figure 3. The survival curves for TC occurrence significantly differed by the duration of radiation exposure. Specifically, the cumulative survival rate of TC decreased with increasing duration of radiation exposure in the hospital.

### Univariate Analysis

Transformation of the KNHS data into a person-period data set yielded 22,759 cases with 105 occurrences of TC. The results of the Kaplan-Meier analysis are summarized in Table 2. Among the sociodemographic variables, age (*χ*^2^_1_=51.6, *P*<.001) and marital status (*χ*^2^_1_=25.1, *P*<.001) were associated with TC occurrence. Among lifestyle variables, sleep problems were significantly associated with TC occurrence (*χ*^2^_1_=20.3, *P*<.001). Among the occupational variables, night shift work (*χ*^2^_1_=20.1, *P*<.001), working units (*χ*^2^_1_=13.0, *P*<.001), and duration of radiation exposure in the hospital (*χ*^2^_1_=91.0, *P*<.001) were associated with TC occurrence.

**Table 2:** Univariate analysis: Kaplan-Meier analysis (n=22,759).

Variables				Participants, n		Event cases, n (%)		Censored cases, n (%)		Log-rank test, *χ*^2^ (*df*)			*P* value		
**Sociodemographic variables**															
	**Age (years)**										51.6 (1)			<.001	
		20-29	17,751		99 (0.6)		17,652 (99.4)								
		≥30	5008		6 (0.1)		5002 (99.9)								
	**Marital status**										25.1 (1)			<.001	
		Married	17,401		93 (0.5)		17,308 (99.5)								
		Unmarried/divorced/separated/widowed	5358		12 (0.2)		5346 (99.8)								
	**Body mass index (kg/m^2^)**										0.004 (1)			.43	
		<23	17,957		79 (0.4)		17,878 (99.6)								
		≥23	4802		26 (0.5)		4776 (99.5)								
**Lifestyle variables**															
	**Smoking**										1.6 (1)			.86	
		Yes	766		1 (0.1)		765 (99.9)								
		No	21,993		104 (0.5)		21,889 (99.5)								
	**Alcohol consumption**										0.5 (1)			.91	
		Yes	15,710		69 (0.4)		15,641 (99.6)								
		No	7049		36 (0.5)		7013 (99.5)								
	**Perceived stress**										0.8 (1)			.83	
		Low (≤11)	21,686		101 (0.5)		21,585 (99.5)								
		High (>11)	1073		4 (0.4)		1069 (99.6)								
	**Sleep problems**										20.3 (1)			<.001	
		Yes (≥12)	1502		14 (0.9)		1488 (99.1)								
		No (<12)	21,257		91 (0.4)		21,166 (99.6)								
**Occupational variables**															
	**Job positions**										3.1 (1)			.87	
		Staff nurse	21,036		99 (0.5)		20.937 (99.5)								
		Nursing unit manager/administrative supervisor	1723		6 (0.3)		1717 (99.7)								
	**Night shift work**										20.1 (1)			<.001	
		Yes	15,746		88 (0.6)		15,658 (99.4)								
		No	7013		17 (0.2)		6996 (99.8)								
	**Working units**										13.0 (1)			<.001	
		General ward/outpatient ward/hospital administration	19,481		84 (0.4)		19,937 (99.6)								
		Special care units^a^	2738		21 (0.8)		2717 (99.2)								
	**Duration of exposure to radiation in hospital**										91.0 (2)			<.001	
		None	18,102		50 (0.3)		18,052 (99.7)								
		<1 year	562		6 (1.1)		556 (98.9)								
		≥1 year	4095		49 (1.2)		4046 (98.8)								

^a^Intensive care unit, emergency room, anesthetic room, and operating room.

### Multivariate Analysis

The results of the Cox regression analysis are presented in Table 3. The value of –2 log-likelihood was 1850.002 (*χ*^2^_1_=65.5, *P*<.001) in the Cox regression model. Among the sociodemographic variables, participants aged in their 20s were significantly more likely to be diagnosed with TC than their older counterparts (HR=4.602, 95% CI 1.893-11.188). Among the occupational variables, those who worked night shifts were more likely to be diagnosed with TC than those who did not work night shifts (HR=1.923, 95% CI 1.127-3.280). In addition, those with radiation exposure in the hospital were more likely to be diagnosed with TC than those not exposed (<1 year: HR=3.449, 95% CI 1.474-8.074; ≥1 year: HR=4.178, 95% CI 2.702-6.461).

**Table 3:** Multivariate analysis: Cox regression analysis (n=22,759).

Variables				Hazard ratio (95% CI)		*P* value
**Sociodemographic variables**						
	**Age (years)**					
		20-29	4.602 (1.893-11.188)		<.001	
		≥30	1.000		—^a^	
	**Marital status**					
		Married	1.000		—	
		Unmarried/divorced/separated/widowed	0.655 (0.345-1.241)		.12	
	**Body mass index (kg/m^2^)**					
		<23	1.000		—	
		≥23	1.416 (0.903-2.222)		.09	
**Lifestyle variables**						
	**Smoking**					
		Yes	0.274 (0.038-1.967)		.17	
		No	1.000		—	
	**Alcohol consumption**					
		Yes	0.759 (0.506-1.140)		.09	
		No	1.000		—	
	**Perceived stress**					
		Low (≤11)	1.000		—	
		High (>11)	0.765 (0.281-2.082)		.25	
	**Sleep problems**					
		Yes (>12)	1.475 (0.832-2.613)		.053	
		No (≤12)	1.000		—	
**Occupational variables**						
	**Job positions**					
		Staff nurse	1.000		—	
		Nursing unit manager/administrative supervisor	0.689 (0.269-1.768)		.44	
	**Night shift work**					
		Yes	1.923 (1.127-3.280)		.03	
		No	1.000		—	
	**Working units**					
		General ward/outpatient ward/hospital administration	1.000		—	
		Special care units^b^	0.754 (0.446-1.275)		.19	
	**Duration of exposure to radiation in the hospital**					
		None	1.000		—	
		<1 year	3.449 (1.474-8.074)		.003	
		≥1 year	4.178 (2.702-6.461)		<.001	

^a^Not applicable.

^b^Intensive care unit, emergency room, and operating room.

## Discussion

### Principal Findings

This longitudinal cohort study of female nurses in South Korea indicated that a longer duration of radiation exposure in the hospital, younger age, and night shift work were significantly associated with the occurrence of TC. These findings suggest that early occupational exposure and circadian disruption play important roles in the development of TC among young women, emphasizing the need for targeted prevention efforts in the health care workforce.

### Comparison With Prior Research

Our results align with findings from major international nursing cohorts. For example, the Nurses’ Health Study II [12] examined the impact of night shift work, sleep disruption, and reproductive factors on TC risk among female nurses in the United States over several decades of follow-up. Similarly, studies from the Danish Nurse Cohort [26] explored the association between occupational exposure, such as job strain, and cancer development, although direct links to TC have not been established. In contrast to these international studies, our investigation specifically focused on young adult nurses in South Korea, a population with one of the highest global incidences of TC, thereby providing important complementary evidence in an under-represented group.

These findings are also consistent with those of a meta-analysis investigating the association between female sex, radiation exposure, younger age, and TC occurrence [27]. Female nurses exposed to radiation in a hospital for more than 1 year had a >4.2-fold higher risk of developing TC than those who did not. This finding is consistent with that of previous large cohort studies on radiation-exposed workers reporting elevated rates of TC and thyroid abnormalities [28]. With the growing use of radiation-based procedures for diagnosis and treatment, occupational radiation exposure is a serious concern for nurses who are often accidentally or unavoidably exposed to radiation. However, as some studies have indicated, thyroid abnormalities related to radiation exposure are often underestimated or under-reported, and low-dose radiation exposure may not always be clearly linked to TC [29]. Chronic low-level exposure to such radiation may have cumulative effects on genetic mutations and rearrangements that contribute to TC development [30], and women tend to be more radiosensitive than men [31]. To reduce the risk of TC development among medical radiation workers in Korea, a regulation requiring a radiation monitoring system and increased radiation protection was enacted, effectively decreasing the occupational radiation dose per calendar year.

Age has long been a key prognostic factor in cancer staging; however, studies have reported inconsistent results concerning the effects of age on TC occurrence. Consistent with our findings, prior studies have revealed that young female adults (age at diagnosis: <30 years) are likely to have a high risk of TC owing to reproductive hormonal changes and high susceptibility to lymph node positivity, which plays an important role in predicting papillary TC recurrence [12]. However, in other Korean studies using a nationwide hospital-based cancer registry, the 30-year to 50-year age group showed the highest TC risk [6]. These inconsistencies underscore the complexity of TC risk factors; however, our findings support the importance of considering earlier TC screening in young adult women.

Univariate analysis indicated that working night shifts and sleep problems were associated with increased TC risk among female nurses. We acknowledge that our cohort of female nurses with sleep problems was shift workers, for whom disruptions in normal circadian rhythms could lead to sleep deprivation [32]. The etiology of sleep disturbances in TC development is not fully understood. However, a possible explanation is that chronic circadian disruption induced by sleep problems plays a role in hypothalamic-pituitary-adrenal axis dysfunction, resulting in tumor development [33]. Nevertheless, sleep disturbance appears to be a common health concern in the general population [34], and previous studies have reported that older women with insomnia or obstructive sleep apnea are at a higher risk of TC [35]. Although our study did not address sleep characteristics in terms of sleep quantity or quality, our findings highlight the importance of circadian health interventions in occupational settings.

In contrast to previous literature suggesting a positive association between obesity and TC risk [15,36], our analysis found no significant link between BMI and TC occurrence. These contradictory findings may be attributed to demographic differences across the study populations. For example, our study sample was limited to female nurses of Korean ethnicity, whereas other studies have examined more diverse racial and ethnic populations. Notably, in a meta-analysis of obesity and TC occurrence, being overweight was associated with a significant increase in TC among non-Asians but not among Asians [36]. Nevertheless, given that overweight or obesity is a major risk factor for TC, additional studies on the association between BMI and TC in Korean women are warranted.

### Strengths and Limitations

This study has several strengths. We leveraged a large-scale, prospective cohort of young female nurses—a medically knowledgeable population known to provide high-quality and reliable self-reported health information. The use of longitudinal data from the KNHS enabled us to examine the effects of time-varying exposures on TC occurrence across multiple survey waves. To appropriately model the temporal relationships between changing exposures and TC risk, we used a longitudinal survival analysis framework using a person-period data structure, which is a widely recommended approach for evaluating time-dependent covariates in event history analyses [37]. Furthermore, this study specifically focused on young adult women aged in their 20s, a high-risk and understudied group in South Korea, contributing novel insights into occupational and lifestyle risk factors for TC development in early life stages.

However, this study has several limitations. First, the KNHS relied on self-reported medical history, such as TC diagnoses, without verification through medical records. Although nurses are generally reliable reporters of health conditions because of their medical knowledge and commitment to research, the possibility of misclassification cannot be entirely excluded [38]. Therefore, the selection of nurses may have enhanced the internal validity of this study. Second, the study did not collect data on TC subtypes or history of benign thyroid diseases, which may have strengthened our assessment of TC risk factors. Third, a detailed quantification of radiation exposure was not possible because radiation-related variables were based on self-reported occupational histories rather than objective dosimetry measurements. Finally, unmeasured confounding factors such as diet, environmental exposure, or detailed sleep characteristics, which could influence the observed associations, were not captured.

### Future Directions

These findings have crucial implications for health care administrators and policymakers. Understanding these risk factors will facilitate informed clinical decision-making and foster a safer working environment. Early thyroid screening protocols for young nurses exposed to radiation or who work night shifts may facilitate early detection and intervention. Hospitals and health care systems should implement rigorous radiation safety programs, provide education on circadian health, and consider scheduling strategies to minimize exposure during long-term shifts. To mitigate the risk of TC, nursing administrators should implement comprehensive risk management strategies for their personnel. Such strategies may encompass regular assessments of both the dose and duration of ionizing radiation exposure, ensuring the availability of appropriate personal protective equipment and facilitating rigorous training on its consistent use. Furthermore, it is essential to inform nurses about the potential TC risks associated with night shift work. Education should also encompass the recognition of sleep deprivation symptoms and recommended interventions when these symptoms manifest.

From a public health perspective, prevention efforts should prioritize TC prevention initiatives targeting young female health care workers, focusing on occupational risk mitigation and health monitoring, starting early in their careers. Future research should aim to quantify cumulative occupational radiation exposure through objective dosimetry; explore dose-response relationships with TC risk; and incorporate detailed sleep measures such as sleep quantity, quality, and circadian chronotype. Such research will help clarify modifiable risk factors and guide the development of evidence-based occupational health guidelines to reduce TC risk in vulnerable populations.

### Conclusion

In this longitudinal cohort study of young female nurses, we identified significant associations between TC occurrence and a longer duration of radiation exposure in the hospital, younger age, and night shift work. These findings underscore the importance of establishing vigilant measures to protect this vulnerable population. In particular, there is an urgent need for consistent monitoring of radiation exposure, especially among younger nurses engaged in nighttime shift duties. Based on these findings, health care institutions should ensure the availability of appropriate personal protective equipment as well as provide comprehensive radiation safety education and circadian health support. These preventive efforts may reduce the occupational burden of TC among young health care professionals.

## Abbreviations

HR: hazard ratio
KNHS: Korea Nurses’ Health Study
STROBE: Strengthening the Reporting of Observational Studies in Epidemiology
TC: thyroid cancer

## Data Availability

The data sets generated or analyzed during this study are available from the corresponding author on reasonable request.
